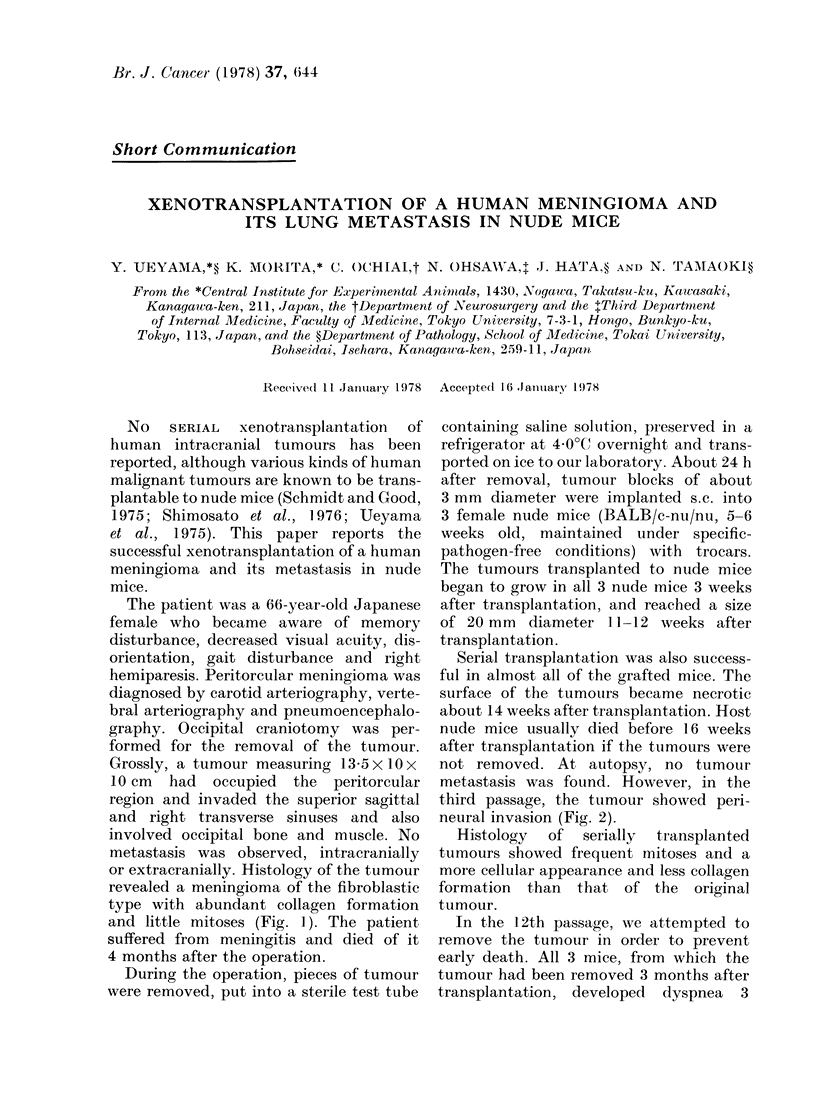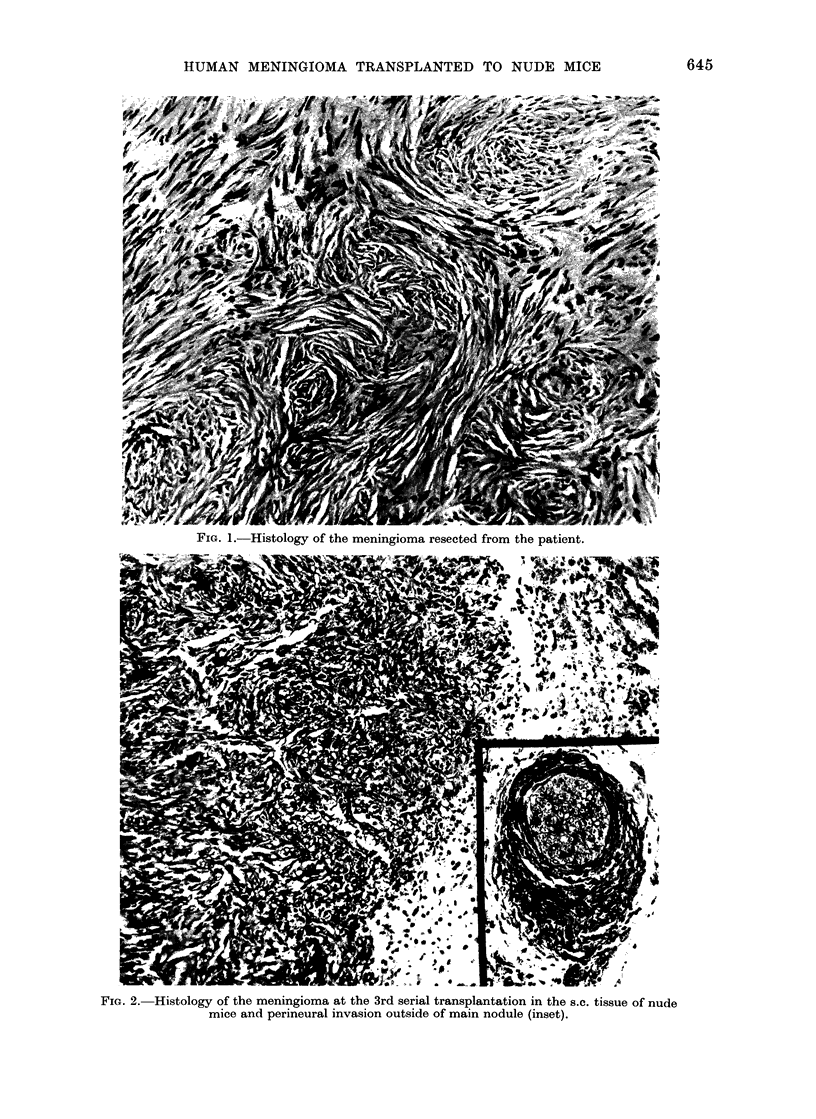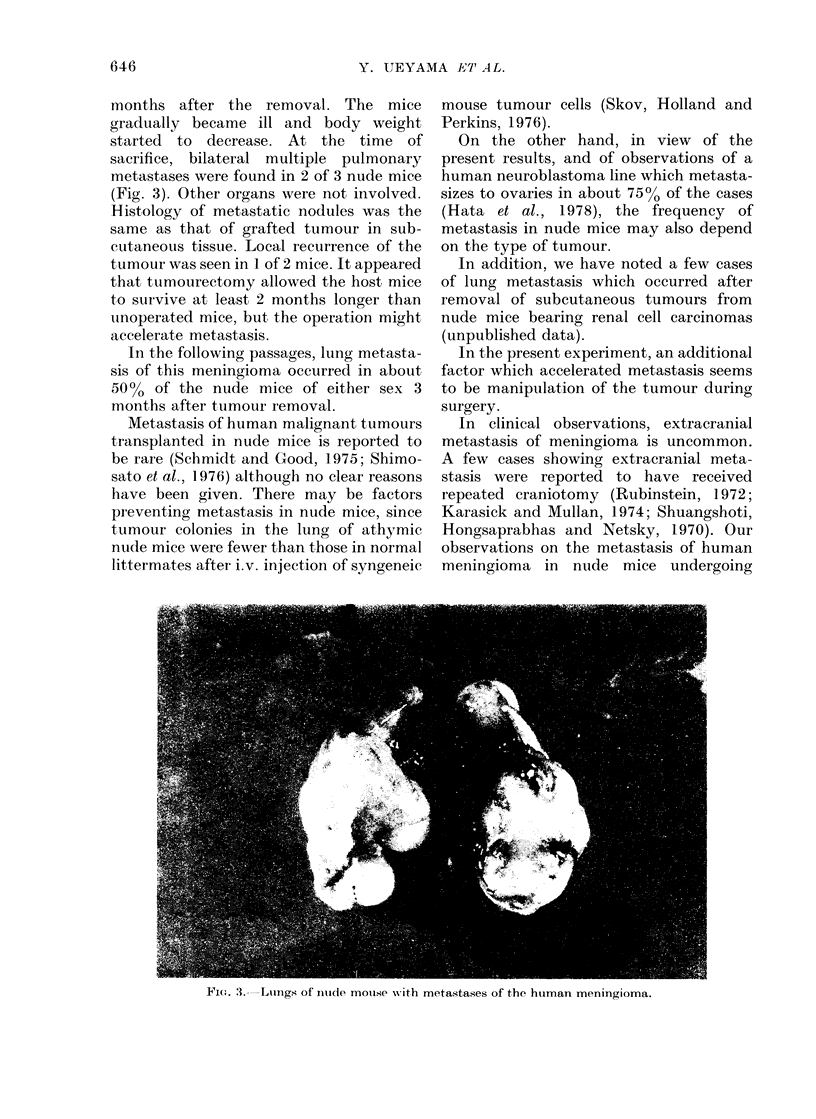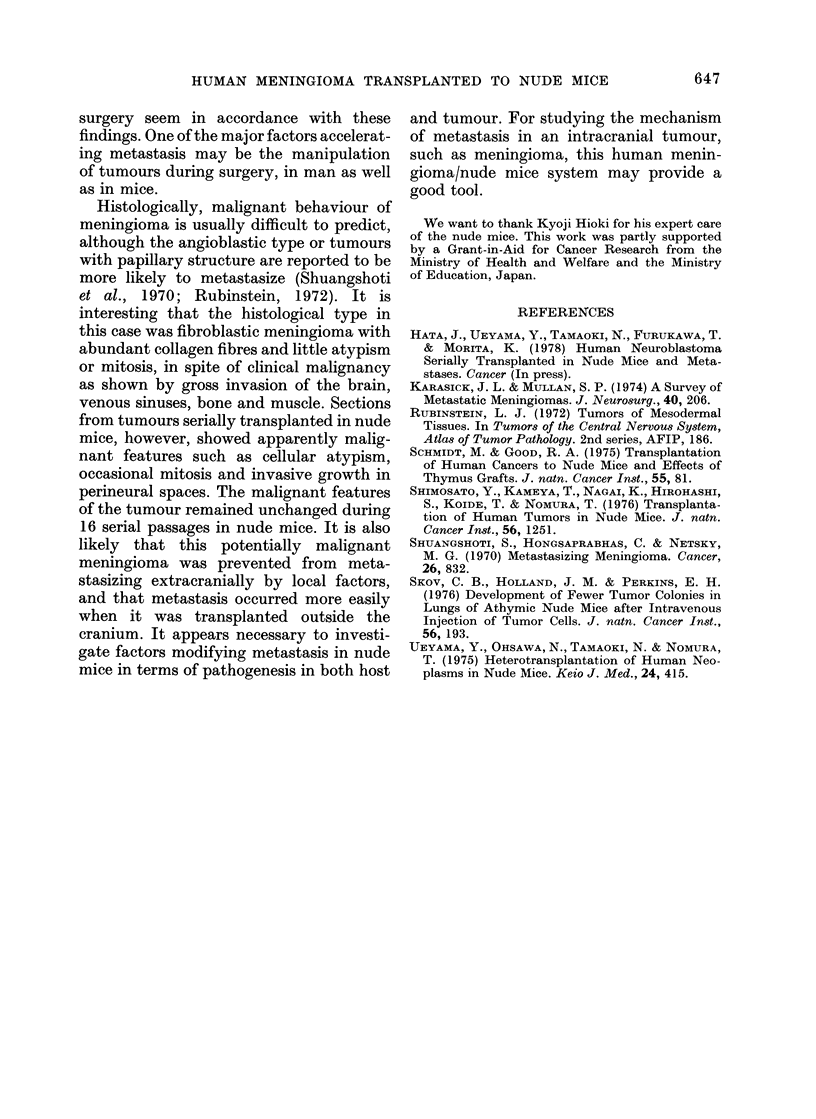# Xenotransplantation of a human meningioma and its lung metastasis in nude mice.

**DOI:** 10.1038/bjc.1978.95

**Published:** 1978-04

**Authors:** Y. Ueyama, K. Morita, C. Ochiai, N. Ohsawa, J. Hata, N. Tamaoki

## Abstract

**Images:**


*Br. J. Cancer* (1978) **37**, 644

***Short Communication***

### XENOTRANSPLANTATION OF A HUMAN MENINGIOMA AND ITS LUNG METASTASIS IN NUDE MICE

Y. UEYAMA,*§ K. MORITA,* C. OCHIAI,† N. OHSAWA,‡ J. HATA,§ AND N. TAMAOKI§

*From the *Central Institute for Experimental Animals, 1430, Nogawa, Takatsu-ku, Kawasaki, Kanagawa-ken, 211, Japan, the †Department of Neurosurgery and the ‡Third Department of Internal Medicine, Faculty of Medicine, Tokyo University, 7-3-1, Hongo, Bunkyo-ku, Tokyo, 113, Japan, and the §Department of Pathology, School of Medicine, Tokai University, Bohseidai, Isehara, Kanagawa-ken, 259-11, Japan*

Received 11 January 1978 Accepted 16 January 1978

NO SERIAL xenotransplantation of human intracranial tumours has been reported, although various kinds of human malignant tumours are known to be transplantable to nude mice (Schmidt and Good, 1975; Shimosato *et al.*, 1976; Ueyama *et al.*, 1975). This paper reports the successful xenotransplantation of a human meningioma and its metastasis in nude mice.

The patient was a 66-year-old Japanese female who became aware of memory disturbance, decreased visual acuity, disorientation, gait disturbance and right hemiparesis. Peritorcular meningioma was diagnosed by carotid arteriography, vertebral arteriography and pneumoencephalography. Occipital craniotomy was performed for the removal of the tumour. Grossly, a tumour measuring $13{\cdot}5 \times 10 \times 10$ cm had occupied the peritorcular region and invaded the superior sagittal and right transverse sinuses and also involved occipital bone and muscle. No metastasis was observed, intracranially or extracranially. Histology of the tumour revealed a meningioma of the fibroblastic type with abundant collagen formation and little mitoses (Fig. 1). The patient suffered from meningitis and died of it 4 months after the operation.

During the operation, pieces of tumour were removed, put into a sterile test tube containing saline solution, preserved in a refrigerator at 4·0°C overnight and transported on ice to our laboratory. About 24 h after removal, tumour blocks of about 3 mm diameter were implanted s.c. into 3 female nude mice (BALB/c-nu/nu, 5–6 weeks old, maintained under specific-pathogen-free conditions) with trocars. The tumours transplanted to nude mice began to grow in all 3 nude mice 3 weeks after transplantation, and reached a size of 20 mm diameter 11–12 weeks after transplantation.

Serial transplantation was also successful in almost all of the grafted mice. The surface of the tumours became necrotic about 14 weeks after transplantation. Host nude mice usually died before 16 weeks after transplantation if the tumours were not removed. At autopsy, no tumour metastasis was found. However, in the third passage, the tumour showed perineural invasion (Fig. 2).

Histology of serially transplanted tumours showed frequent mitoses and a more cellular appearance and less collagen formation than that of the original tumour.

In the 12th passage, we attempted to remove the tumour in order to prevent early death. All 3 mice, from which the tumour had been removed 3 months after transplantation, developed dyspnea 3

FIG. 1.—Histology of the meningioma resected from the patient.

FIG. 2.—Histology of the meningioma at the 3rd serial transplantation in the s.c. tissue of nude mice and perineural invasion outside of main nodule (inset).

months after the removal. The mice gradually became ill and body weight started to decrease. At the time of sacrifice, bilateral multiple pulmonary metastases were found in 2 of 3 nude mice (Fig. 3). Other organs were not involved. Histology of metastatic nodules was the same as that of grafted tumour in subcutaneous tissue. Local recurrence of the tumour was seen in 1 of 2 mice. It appeared that tumourectomy allowed the host mice to survive at least 2 months longer than unoperated mice, but the operation might accelerate metastasis.

In the following passages, lung metastasis of this meningioma occurred in about 50% of the nude mice of either sex 3 months after tumour removal.

Metastasis of human malignant tumours transplanted in nude mice is reported to be rare (Schmidt and Good, 1975; Shimosato *et al.*, 1976) although no clear reasons have been given. There may be factors preventing metastasis in nude mice, since tumour colonies in the lung of athymic nude mice were fewer than those in normal littermates after i.v. injection of syngeneic mouse tumour cells (Skov, Holland and Perkins, 1976).

On the other hand, in view of the present results, and of observations of a human neuroblastoma line which metastasizes to ovaries in about 75% of the cases (Hata *et al.*, 1978), the frequency of metastasis in nude mice may also depend on the type of tumour.

In addition, we have noted a few cases of lung metastasis which occurred after removal of subcutaneous tumours from nude mice bearing renal cell carcinomas (unpublished data).

In the present experiment, an additional factor which accelerated metastasis seems to be manipulation of the tumour during surgery.

In clinical observations, extracranial metastasis of meningioma is uncommon. A few cases showing extracranial metastasis were reported to have received repeated craniotomy (Rubinstein, 1972; Karasick and Mullan, 1974; Shuangshoti, Hongsaprabhas and Netsky, 1970). Our observations on the metastasis of human meningioma in nude mice undergoing

FIG. 3.—Lungs of nude mouse with metastases of the human meningioma.

surgery seem in accordance with these findings. One of the major factors accelerating metastasis may be the manipulation of tumours during surgery, in man as well as in mice.

Histologically, malignant behaviour of meningioma is usually difficult to predict, although the angioblastic type or tumours with papillary structure are reported to be more likely to metastasize (Shuangshoti *et al.*, 1970; Rubinstein, 1972). It is interesting that the histological type in this case was fibroblastic meningioma with abundant collagen fibres and little atypism or mitosis, in spite of clinical malignancy as shown by gross invasion of the brain, venous sinuses, bone and muscle. Sections from tumours serially transplanted in nude mice, however, showed apparently malignant features such as cellular atypism, occasional mitosis and invasive growth in perineural spaces. The malignant features of the tumour remained unchanged during 16 serial passages in nude mice. It is also likely that this potentially malignant meningioma was prevented from metastasizing extracranially by local factors, and that metastasis occurred more easily when it was transplanted outside the cranium. It appears necessary to investigate factors modifying metastasis in nude mice in terms of pathogenesis in both host and tumour. For studying the mechanism of metastasis in an intracranial tumour, such as meningioma, this human meningioma/nude mice system may provide a good tool.

We want to thank Kyoji Hioki for his expert care of the nude mice. This work was partly supported by a Grant-in-Aid for Cancer Research from the Ministry of Health and Welfare and the Ministry of Education, Japan.

### REFERENCES

HATA, J., UEYAMA, Y., TAMAOKI, N., FURUKAWA, T. & MORITA, K. (1978) Human Neuroblastoma Serially Transplanted in Nude Mice and Metastases. *Cancer* (In press).

KARASICK, J. L. & MULLAN, S. P. (1974) A Survey of Metastatic Meningiomas. *J. Neurosurg.*, **40**, 206.

RUBINSTEIN, L. J. (1972) Tumors of Mesodermal Tissues. In *Tumors of the Central Nervous System, Atlas of Tumor Pathology*. 2nd series, AFIP, 186.

SCHMIDT, M. & GOOD, R. A. (1975) Transplantation of Human Cancers to Nude Mice and Effects of Thymus Grafts. *J. natn. Cancer Inst.*, **55**, 81.

SHIMOSATO, Y., KAMEYA, T., NAGAI, K., HIROHASHI, S., KOIDE, T. & NOMURA, T. (1976) Transplantation of Human Tumors in Nude Mice. *J. natn. Cancer Inst.*, **56**, 1251.

SHUANGSHOTI, S., HONGSAPRABHAS, C. & NETSKY, M. G. (1970) Metastasizing Meningioma. *Cancer*, **26**, 832.

SKOV, C. B., HOLLAND, J. M. & PERKINS, E. H. (1976) Development of Fewer Tumor Colonies in Lungs of Athymic Nude Mice after Intravenous Injection of Tumor Cells. *J. natn. Cancer Inst.*, **56**, 193.

UEYAMA, Y., OHSAWA, N., TAMAOKI, N. & NOMURA, T. (1975) Heterotransplantation of Human Neoplasms in Nude Mice. *Keio J. Med.*, **24**, 415.